# Lived experience of comfort with sexuality and fertility for survivors of hematopoietic progenitor cell transplants: phenomenological study

**DOI:** 10.1007/s11764-025-01752-1

**Published:** 2025-02-18

**Authors:** Lúcia Bacalhau, Patrícia Pontífice-Sousa

**Affiliations:** Center for Interdisciplinary Research in Health, Faculty of Health Sciences and Nursing, Universidade Cátolica Portuguesa, Palma de Cima, 1649-023 Lisbon, Portugal

**Keywords:** Comfort, Survivor, Sexuality, Fertility, Stem cell transplant, Lived experience, Nursing, Phenomenology

## Abstract

**Introduction:**

Sexuality and fertility are deeply personal and fundamental aspects of human identity and quality of life. For survivors of hematopoietic stem cell transplants (HSCT), these areas can be profoundly affected by the physical, emotional, and social impacts of the treatment. Discomfort or lack of clarity regarding these issues can lead to long-term psychological distress, relationship challenges, and decreased overall well-being. Addressing this discomfort through research helps to fill a critical gap in supportive care, providing survivors with the tools and knowledge they need to navigate these challenges and enhance their post-treatment quality of life.

**Purpose:**

To grasp the scope of sexuality and fertility within the lived experiences of comfort and discomfort among allogeneic stem cell transplant survivors.

**Methods:**

We employed a qualitative approach through van Manen’s phenomenology of practice. The phenomenon was revealed via phenomenological interviews, incorporating narratives and illustrative episodes that captured the lived experiences of 20 survivors. We gathered descriptions of these experiences from participants from July 2020 to May 2021. During our phenomenological reflection on these accounts, we adhered to stages like epoché, reduction, and vocative, as outlined by van Manen (van Manen 2014).

**Results:**

The exploration of comfort within the realms of sexuality and fertility yielded several themes: desire and the relationship; immunosuppression as a limitation on sexual activity; the body does not correspond to desire; the future: fertility and the couple’s relationship; the challenges of disclosure and intervention.

**Conclusion:**

The findings indicate that survivors have long-term challenges with sexual expression and activity, stemming from the physical constraints of graft-versus-host disease (GVHD), perceptions of their immunosuppression, and the significance of their partnership, all of which translate into requirements for nursing care.

**Implications for Care and Support Cancer Survivors:**

Considering the experiences of these participants and the observed comfort linked to the sexuality of ASCT survivors, we can contemplate the care practices and recognize sexuality as a field of action for nurses in this context to enhance comfort for these individuals.

## Introduction

Allogeneic stem cell transplantation (ASCT) offers a realistic prospect of curing oncological diseases or permanently addressing chronic hematological, immunological, or metabolic disorders. However, it is a lengthy process fraught with changes and complications stemming from the individual’s condition, treatment, and even family, social, and community factors that may affect their well-being. Those undergoing ASCT experience a multifaceted process filled with unique sensations, emotions, thoughts, and experiences, presenting a challenge to healthcare due to their specialized and extended care needs [1]. The emergence of cancer survivors as a distinct group with particular needs, owing to the after effects or complications of their treatments, is now acknowledged [2, 3]. With early cancer detection and more effective treatments, the trajectory of the disease has shifted, resulting in a growing population of individuals who survive cancer and live on for many years post-diagnosis [4].

Recognizing that each individual experiences situations and circumstances uniquely, we have embraced the definition of a survivor by Carlyle et al. [5] which considers a person who has finished treatment and is in remission. Survivors of ASCT live with chronic conditions, facing multiple discomforts due to lifestyle changes, altered expectations, life plans, meanings, distress, and the adoption of specific therapeutic regimens. Given this reality, and considering the holistic functioning of humans, it becomes crucial to address the complete pattern of the individual’s life process, particularly in how they perceive comfort [6]. Comfort is simultaneously a noun, verb, adjective, a state, a process, and an outcome [7]. Despite its complexity, comfort is essential in holistic human care, a fundamental concept in nursing, and a vital part of the care process [7, 8].

Research interest in the concept of comfort in nursing has been growing [7, 8]. Kolcaba categorizes comfort into three types: relief, ease, and transcendence The concept of “comfort” is multifaceted, encompassing physical, emotional, psychological, and social dimensions. At its core, comfort can refer to relief from pain or distress, a sense of ease and security, or the presence of supportive environments that promote well-being. In healthcare, comfort is often positioned as a fundamental therapeutic goal, reflecting its importance not only in alleviating suffering but also in enhancing the overall quality of life. However, the meaning and significance of comfort can vary greatly depending on individual experiences, cultural contexts, and the specific challenges faced [7, 8].

In the context of survivors of hematopoietic stem cell transplants (HSCT), comfort takes on a unique and critical importance. These individuals often experience profound physical changes, such as hormonal disruptions and infertility, alongside emotional and psychological challenges, including shifts in identity and self-perception. Sexuality and fertility, as deeply personal aspects of life, can become sources of significant discomfort, marked by uncertainty, fear, and feelings of loss. For survivors, achieving comfort in these areas involves not only physical recovery but also emotional adjustment, renegotiation of relationships, and integration of new self-understandings [9–11].

Existing literature underscores the pivotal role of comfort in healthcare, particularly for individuals coping with chronic illness or recovering from invasive treatments. Studies have shown that comfort can enhance emotional resilience, foster a sense of agency, and improve adherence to medical interventions. In the realm of sexuality and fertility, comfort is closely linked to open communication, access to supportive resources, and the validation of individual experiences. Research also highlights the barriers to achieving comfort, including stigma, lack of information, and inadequate support systems [10].

The sexual and emotional aspects of a person’s life are significantly affected following an allogeneic hematopoietic stem cell transplant (ASCT). Often, this issue is overlooked by both patients and caregivers. The myriad of physical, endocrine, and genital complications associated with chronic graft-versus-host disease (cGVHD) are complexly linked with psychological disorders [9, 10, 12].

By exploring comfort within the lived experiences of ASCT survivors, this study seeks to illuminate how individuals navigate these complex challenges. It examines how comfort—or the absence of it—shapes survivors’ perceptions of themselves, their relationships, and their futures. This focus on comfort not only contributes to a deeper understanding of survivors’ needs but also provides valuable insights for healthcare providers aiming to deliver holistic, patient-centered care.

The impact of this situation on personal, familial, and social levels is acknowledged, as well as the accompanying instability and insecurity. Despite understanding that the severity of complications and necessary life adjustments in intimacy and sexuality can affect comfort during transplantation and survival, the actual experience of comfort in this domain remains unexplored.

Investigating the meaning individuals assign to their experiences helps identify their needs and provide appropriate responses. Since the interpretation of experiences varies from person to person, grasping the daily reality of survivors and considering the key aspects that define the concept of comfort is crucial.

The focus of the research conducted centers on exploring the lived experiences of comfort and discomfort related to sexuality and fertility among survivors of hematopoietic stem cell transplants. This phenomenological study seeks to understand how these individuals navigate the physical, emotional, and relational challenges tied to these aspects of their lives. In the subsequent sections, the manuscript will provide a detailed overview of the study’s methodology, present key findings from participant narratives, and discuss these findings in relation to existing literature. Finally, it will offer practical recommendations for improving supportive care and addressing the specific needs of this population.

## Methods and design

### Methodology

To grasp the lived experience of comfort, we employed a qualitative methodology based on van Manen’s (2014) phenomenology of practice [11, 13]. This type of phenomenology is deeply rooted in specific epistemological and ontological stances that prioritize the exploration of subjective experience. Epistemologically, phenomenology emphasizes understanding the world as it is perceived and experienced by individuals, acknowledging that knowledge arises from lived realities rather than detached observation. Ontologically, it foregrounds the interconnectedness of mind and body, rejecting Cartesian dualism and instead embracing a holistic perspective where physical and emotional experiences are inseparable.

Central to this framework are concepts such as “lived experience” and “lived body,” which encapsulate the idea that our understanding of existence is mediated through our embodied presence in the world. The “lived experience” acknowledges the subjective, first-person perspective as a valid and essential source of insight, while the “lived body” highlights the body not merely as an object but as a site of experience, sensation, and meaning [14, 15]. These ideas are particularly significant in contexts like sexuality and fertility after medical interventions, where individuals’ embodied experiences deeply influence their sense of self and quality of life.

Exploring these philosophical foundations enriches the study by situating the experiences of hematopoietic stem cell transplant survivors within a broader understanding of human existence, providing a lens to examine how individuals perceive, interpret, and navigate their realities. Van Manen’s phenomenological approach is grounded in the interconnected concepts of epoché, reduction, and vocative, which together facilitate a deep exploration of lived experience. Epoché involves the suspension of preconceived notions and biases, allowing the researcher to engage with the phenomenon authentically, free from prior assumptions. Complementing this, reduction focuses on uncovering the essential structures of the experience by stripping away extraneous elements, ensuring that the inquiry remains centered on the core meanings. Finally, the vocative aspect emphasizes the evocative power of phenomenological writing, using expressive and poetic language to resonate deeply with readers, calling forth their own sense of the experience. Together, these tools enable researchers to illuminate the richness and depth of human experiences, presenting them in a way that is both intellectually and emotionally compelling [11, 13].

### Theoretical framework

Comfort, being a broad and multifaceted concept, requires elucidation within the lived experiences of ASCT survivors. Therefore, exploring these experiences through phenomenology can lead to insights that promote equitable, respectful, and comforting care. Phenomenology, as a philosophical method for examining experience, is a human science focused on the meaningful structure of lived experiences. Hermeneutic significance is bestowed upon these experiences when they are reflected upon and imbued with memory [16].

### Setting and participants

The study involved individuals who had received allogeneic stem cell transplants and were under outpatient follow-up. Eligible participants were those who (i) were aged 18 or older; (ii) had been survivors and undergone ASCT for a minimum of three months; (iii) showed no signs of disease relapse; (iv) could verbally communicate, providing information pertinent to the study, and articulate their emotions and feelings.

The participants were recruited in person from the day hospital of a bone marrow transplant unit. They were informed about the study’s objective and were only included after receiving clarification and signing the informed consent.

A total of twenty individuals shared their experiences as survivors for this research (Table 1). The participant count was determined with the aim of gathering detailed accounts of personal experiences to form experiential examples, revealing the essence of these experiences. Sufficient narratives were obtained to create a representative and coherent structure that addressed the central research questions [17]. The collection of experiences ceased upon reaching 20 participants, at which point the narratives provided a rich, intricate, and nuanced understanding of the experiences, unveiling a previously unexplored realm.

**Table 1:** Characterization of the participants

Survivor name (fictitious)	Age	Sex	Diagnose	Years after ASCT	Presence of GVHD
Isabel	52	Female	Monomac syndrome	4	yes
Amélia	43	Female	Acute myeloid leukemia	5	yes
Alice	44	Female	Non-Hodgkin lymphoma	5	yes
João	45	Male	Acute myeloid leukemia	5	yes
Anita	33	Female	Acute myeloid leukemia	2	No
Camila	40	Female	Non-Hodgkin lymphoma	2	No
Santiago	43	Male	Non-Hodgkin lymphoma	2	Yes
Rodrigo	50	Male	Acute lymphoblastic leukemia	4	Yes
Carmo	41	Female	Acute lymphoblastic leukemia	4	Yes
Afonso	58	Male	Acute myeloid leukemia	3	Yes
Mafalda	42	Female	Hodgkin disease	12	Yes
Matilde	49	Female	Hodgkin disease	1	No
Tiago	60	Male	Non-Hodgkin lymphoma	8	Yes
Diana	45	Female	Acute myeloid leukemia	2	Yes
Sara	44	Female	Acute myeloid leukemia	2	No
Diogo	32	Male	Acute lymphoblastic leukemia	9	Yes
Francisco	49	Male	Myelodysplastic syndrome	5	Yes
Miguel	50	Male	Chronic myeloid leukaemia	1	No
Raquel	44	Female	Acute lymphoblastic leukemia	10	Yes
Duarte	60	Male	Acute lymphoblastic leukemia	3	Yes

## Data collection

Following van Manen’s [18] guidelines, the principal investigator conducted a phenomenological interview in which participants were asked to concentrate on a specific experience. They were encouraged to describe the event in as much detail as possible, focusing on their feelings, mood, and emotions. Special attention was given to how sensations were experienced, such as “what sounds were felt,” guiding the survivor to reflect on and emphasize what was lived during that moment, as though they were returning to the experience itself.

The interviews were carried out in a place chosen by the survivor, guaranteeing the person’s privacy, in a comfortable space: at home, in a garden, at the hospital, on a terrace. The interviews lasted between 1 and 2 h in one moment.

In an approximation to reality, we also used the collection of illustrative material from sources such as literature, in this case, literature written by the survivors themselves, of the participants’ choice, which served as catalysts for reflection, in order to illustrate and expose aspects that further elucidated the phenomenon, clarifying it [14]. After completing the transcription, it was returned to each survivor to seek validation of the researchers’ interpretations. They validated the transcription and added information—experiences that were lived in order to complement the lived experience descriptions.

## Data analysis

To describe the lived experience of ASCT survivors, we identified the themes that emerged from the lived experience descriptions. In this process, we sought to capture the themes “by removing appropriate phrases or capturing in simple statements the core meaning of the themes” as suggested by van Manen ([11] p. 93). The process used to identify the themes of the phenomenon under study was developed in four moments: (i) first, after the transcription and compilation of all the lived experience descriptions, a holistic reading was performed so as to capture the meaning and sense of the whole (van Manen, 1990, 2014); (ii) then a detailed and reflective reading of the descriptions was performed, where we tried to identify the essential and revealing phrases of the lived experience under study; (iii) in a third moment, the thematic units extracted from the descriptions that show essential themes related to the phenomenon were highlighted. The essential themes allowed guidelines for the discovery of the major dimensions of the lived experience and “make the phenomenon what it is, and without which the phenomenon could not be what it is” [11]. At this stage, looking at all the sentences and group of sentences was guided by the question: what do these sentences or group of sentences reveal of the lived experience described? We used software Maxqda to support the discovery of the themes.

From here emerged the relationship with the themes and subsequent construction of the text illustrating the results of the study. We do not attribute meaning to the number of occurrences of each experience; an experience had relevance to construct the final meaning picture. Throughout the phenomenological reflection, trustworthiness and confirmability were ensured by involving experts familiar with qualitative research [11, 13]. Their input helped validate the analysis process and interpretations, providing an external perspective that reinforced the credibility of the findings. This expert involvement served to confirm that the research was conducted rigorously and that the conclusions drawn accurately reflected the participants’ lived experiences. (iv) Next, we proceeded to phenomenological writing with the objective of constructing a text evoking the description of human actions, behaviors, intentions, and experiences as they are uncovered. Given that the aim was to build an evocative text that accessed the experience as it is lived by people, it was important to use sources that allowed illustrating the meaning of the phenomenon under study, shared by some of the participants [13].

### Ethical considerations

Before each description of lived experiences was collected, participants completed a free and informed consent form, having read it and resolved any uncertainties. The research process ensured the complete confidentiality of the participants. The collected data was de-identified, and a pseudonym was assigned by the first author. The data was securely stored in an encrypted digital system, and only the researchers had access. All data that was not necessary for future publication was destroyed. They were informed of their right to withdraw from the study at any time without affecting their care. The Research Ethics Committee of the Instituto Português de Oncologia de Lisboa, Francisco Gentil EPE (UIC Code 1314), approved this research, which adhered to the Declaration of Helsinki (1964). The research upheld confidentiality and the principles of beneficence and non-maleficence, in line with the EQUATOR communication standards.

## Results

The experience of intimacy and sexuality is a vital component of a survivor’s sense of comfort, deeply influenced by the course of illness, treatment, and the subsequent period of survival. The process of ASCT can impact various aspects of the intimate and sexual realm.

### Desire and the relationship

Throughout the illness and treatment phase, a decrease or absence of sexual activity is commonly reported. Individuals often do not feel capable of engaging in sexual activity, lacking the necessary physical, psychological, and emotional resources to incorporate it into their lives. In the survival phase, individuals attempt to reclaim the aspects of sexuality that were suppressed, becoming cognizant of new challenges and restrictions. It is noted that libido often diminishes during the illness and may persist even after treatment has concluded. The path to regaining libido is multifaceted, extending beyond hormonal changes, and profoundly affects self-esteem and self-image, thereby influencing the living situation and the dynamics within the partnership. Afonso, Alice, and Anita said:

Afonso: “*Indeed, there has been a change in sexuality, and it remains unresolved. My libido has significantly decreased! For a long stretch, and even now, it’s inconsequential; it doesn’t cross my mind, it’s absent. Occasionally, there’s a flicker, but it’s not a part of my life. It’s not particularly troubling to me, but it does affect my relationships, which I consider occasionally. It’s a profound side effect, indeed*.”

Alice: “*Considering your intimate life, I understand there are alternatives to sex, which remains significant. The sex life is still out of sync, and the relationship suffers from this disconnection*.”

Anita: “*I’ve observed a decline in sexual activity compared to before the illness. However, it seems to be a gradual change because my libido is now improving*.”

The dynamics between partners and their roles during an illness significantly influence the resumption of sexual activity, and this is interpreted uniquely by each couple. The continuous presence and the shift from being a partner to a caregiver can complicate the return to sexual intimacy. Raquel and Santiago said:

Raquel: “*My husband and I became mere shadows, remnants of the young, joyful, and hopeful couple we once were (…) his primary role evolved into that of a ‘carer’ (…) he was more than my spouse; he became akin to my father and brother*.”

Santiago: “*It has changed. It has changed because being together constantly makes things more complex. For instance, when we tire of something, we take a walk, and it dissipates, right? Since we’re not separated all day due to work, our dynamic shifts. That feeling of ‘nostalgia,’ right? Nostalgia drives our desire to be together. While it has, on one hand, brought us closer, on the other, it has also made us too comfortable and, paradoxically, more distant in a way. It’s more of the same, essentially!*”

On the other hand, her partner stepping back from the caregiver role hindered the growth of sexuality and intimacy within another couple’s relationship. Afonso experienced, “*After such a long absence of contact, it’s challenging to reconnect. Thankfully, beyond the initial shock of my wife no longer being a caregiver, there are other forms of intimacy and strong bonds that compensate for the losses. Time will tell what unfolds* (…).”

This process of regaining sexual desire involves more than just hormonal changes; it profoundly impacts self-esteem, self-image, and partnership dynamics. The shift in roles during illness, from partner to caregiver, can complicate the return to intimacy, with some couples struggling to reconnect sexually. However, for others, different forms of intimacy help maintain strong bonds, though the path to restoring a fulfilling sexual relationship remains complex and unique to each couple.

### Immunosuppression as a limitation on sexual activity

Participants shared their experiences of prolonged immunosuppression, describing a sense of vulnerability to disease and its effect on their sexual and intimate experiences as individuals and as couples. The fear of infection, the imperative to avoid illness, and the necessity of maintaining infection control post-transplant shape their sexual lives. The COVID-19 pandemic has intensified these fears due to the increased risk of contagion, with partners often being more exposed as they continue to shop, work, and perform other activities outside the home. Afonso explained: “*With the pandemic, the inability to kiss for an extended period, especially post-transplant, has compounded over time. Even contemplating a simple, light kiss raises the question, ‘Can I?’ The absence of such contact makes it difficult to reengage.*” Camila felt the same, “*I strive to provide her with all the love and pleasure possible, despite the constraints of our current situation. It’s been a year and a half, and I’m still not ready to forego any form of protection*.”

Participants shared how prolonged immunosuppression made them feel vulnerable to disease, significantly affecting their sexual and intimate experiences, both individually and as couples. The need to avoid illness and maintain infection control post-transplant shaped their sexual lives, and the COVID-19 pandemic heightened these fears, especially as partners were more exposed due to activities outside the home.

### The body does not correspond to desire

Physical pain and mobility limitations act as barriers to sexuality within the couple’s relationship. The discomfort linked to osteonecrosis and the cutaneous and articular manifestations of GVHD complicates movement and restricts these individuals from participating in sexual activities. Carmo shares: “*It’s been non-existent for a long time. Walking is a struggle, as is simply turning over in bed. With the lockdown, since he goes to work, our time together is minimal. We’re hardly ever close; he sleeps in the office, and I sleep in the bedroom, sometimes with my daughter. The lack of intimacy has persisted since my pneumonia in 2019. The last occurrence was some time back, following my hip surgery. There was a phase before my diagnosis when intimacy ceased, and my extended stays in Lisbon also contributed to that absence. Post-surgery, I was on the path to recovery, but then regressed. The pneumonia, the eight months in the hospital, and my complete immobility took their toll. Now, even with slight movement, pain is a constant. I deeply miss the intimacy, but I am at a loss for what to do*.” Francisco shared, “(…) *I experience significant pain at night. Any movement or touch to my legs causes pain. Consequently, I've started sleeping in a different room. We haven’t shared a bed since*.”

Changes in lubrication are noticeable, with mucosal dryness resulting from treatments or the onset of menopause leading to discomfort and restricting sexual activity. Sara noticed: “*Initially, we observed that my skin was extremely dry, which I believe is due to the chemotherapy. However, our desire to be together never waned*.” Raquel felt “*Lubrication is altered, I felt dry, no matter how much stimulation I had and this consequently caused pain during sexual activity*.”

Sexual impotence, in the case of men, limits the fulfillment of sexual activity and causes discomfort, given the impact this limitation has on self-image and self-concept. It interferes with the survivor’s sense of masculinity. João said: “*It’s changed, it’s changed. It’s changed, and just yesterday I went to the endocrinologist and reinforced this again with the doctor. There was a time when I couldn’t get an erection. I take the intimacy part to mean sexuality, because all the other intimate parts are impeccable. There was a long period after the transplant when I couldn’t.(…) I have to admit that I’ve never been the same again in that respect, unfortunately. Although the medication continues to help, unfortunately there are things that haven’t gone back to the way they were before. It’s like anything, sometimes it goes well, sometimes it goes badly, sometimes not so well, sometimes we just stop there*.” Tiago revealed, “*I’ve tried to resume sexual activity, it’s part of life, isn’t it? A man wants to feel capable of fulfilling a relationship. I’ve been undergoing treatments to regain my erectile capacity, but it’s not easy*.”

Physical pain and mobility limitations due to osteonecrosis and GVHD complicate intimacy for couples, as individuals struggle with movement and discomfort during sexual activity. Additionally, changes in lubrication, sexual impotence, and the emotional toll of these challenges, such as concerns about self-image and masculinity, further hinder sexual relationships and intimacy, with some participants expressing a deep longing for closeness despite these obstacles.

### The guilt of not matching their partner

The wish to resume or sustain an active sexual life often clashes with physical limitations, libido, and self-image issues, causing guilt in some survivors. This guilt stems from the perception of not fulfilling their role in the relationship or meeting their partner’s expectations regarding intimacy and sexuality. Alice shared, “*I’m not hesitant to discuss these matters, to acknowledge the tremendous challenge of intimacy. (…) But if it weren’t for my husband, who looks beyond, it would be incredibly hard to cope. I might even describe it as a sense of guilt, despite knowing deep down that I’m not at fault. It’s feasible to claim innocence yet still feel guilty. It’s feasible! I never chose to have lymphoma, to enter menopause, which I have, or to develop osteoporosis, nor any of the issues that typically come after 60, which are exacerbated at 40 or even in my 30s, with all the complications they bring to one’s intimate life*.” Diana adds, “*It’s truly challenging because it involves aspects of the relationship that aren’t central. Understanding is key, recognizing our frailties, acknowledging that we wake up at night craving sleep. We need rest. Communication always helps. We seek respect and must also comprehend the other’s perspective, striving to reconcile as soon as possible. (…) Availability isn’t always present, yet we learn and must exhibit determination. It’s not always to our liking, but we endeavor to accommodate without compromising ourselves. Personally, I value these situations more, having experienced them during breastfeeding, and now, I shed tears when I think ‘I might not have been here.’ It matters to both parties, and fortunately, it signifies that the passion persists. Keeping the flame alive through life’s challenges is never simple*.”

GVHD can manifest in the vaginal mucosa, resulting in vaginal stenosis that impedes penetration and may lead to discomfort. Those who endure this condition frequently report a reduced sense of femininity. It significantly impacts current relationships and obstructs the development of new ones, affecting their sense of fulfillment as women. Amélia shared, “*The gynecological graft hinders my ability to have sex. It bars me from initiating a relationship because I am unable to engage in sexual activity. This makes me feel less of a woman, which is the most devastating aspect. I suffer from stenosis. The condition used to cause me daily pain, but that has ceased. Now, the one thing I am unable to do is have sex*.” Isabel revealed, “*I feel embarrassed to admit it, but it significantly impacts me intimately. Since my transplant, graft-versus-host disease has affected my mucous membranes, particularly my vagina. I have hypertrophy and am unable to have sexual intercourse—it’s as if I’ve become a virgin again. It’s quite obstructive in that sense. Additionally, I’ve had some difficulty discussing these issues with my doctor due to embarrassment. My husband, being older, doesn't seem too concerned. He’s actually more at ease now, thinking, ‘I’m old, she’s young, and that’s just how it is.’ He used to be very suspicious and jealous, but now he’s calmer, yet I still feel uneasy*.”

The desire to resume or maintain an active sexual life is often hindered by physical limitations, changes in libido, and concerns about self-image, leading some survivors to experience guilt over not meeting their partner’s expectations. Additionally, conditions like GVHD can result in vaginal stenosis and discomfort, further impacting intimacy and self-esteem, with some survivors feeling less feminine and facing challenges in both current and potential future relationships.

### The future: fertility and the couple’s relationship

Fertility concerns are significant for some survivors, as infertility may result from their treatments. After completing treatment, when they feel ready, the possibility of having children is a concern, and infertility confirmation can bring sadness. Camila felt, “*I would like to give my son a sibling, but I doubt that’s possible, and it’s uncomfortable discussing it with friends. It’s a realization that comes later; had I known what I know now, I would have chosen to have children earlier. The decision to focus on my career or to have children was based on my career in business, but in hindsight, having children earlier would have been better. I was younger, had more energy to care for a child, to play, and to enjoy the activities that bring me joy without the fatigue from illness or life’s demands. My son could have had a sibling to share experiences with, but currently, he only has his father and mother*.” João expresses deep regret, lamenting that X., in her previous relationship, chose not to become a mother as it did not resonate with her. He mourns the lost opportunity, especially since she had expressed a desire for it, and he is saddened by her infertility. At this juncture in the survivor’s journey, the choice to safeguard fertility prior to initiating treatment is reconsidered. There is a sense of sorrow over the inability to maintain the option of parenthood. João shares, “*When I was admitted to the hospital, Dr. X. informed me on a Friday that it was too late; we couldn’t delay until Monday for me to go to the MAC for preservation. It was truly unfortunate, a significant loss, because I had postponed it with the intention to proceed, as it was absolutely logical, given that X. would have been an extraordinary mother*.”

Understanding the impact of the process on the endocrine system is necessary to adjust life expectations concerning the menstrual cycle and reproductive function. Anita shares, “*The concern is not fertility but menstruation. I’m uncertain if I will recover; it’s not confirmed whether my ovaries are still functional. This worries me—not the infertility, since I was infertile before the cancer, but because, as a thirty-two-year-old woman, hormonal regulation through my menstrual cycle is important. If it doesn’t resume, there could be other consequences. My doctor advised taking the pill to regulate my endometrium, yet ovarian failure hasn’t been ruled out. Honestly, the most disheartening aspect is the inability to have children, although that was the case even before the cancer. Nevertheless, I can still become a mother*.” Matilde expressed, “*Everything has changed, everything has changed. It’s only now that I’m seeing an endocrinologist; I’m uncertain if I’m menopausal. All I know is that my periods have stopped for years, and I’m unsure if it’s a result of the chemotherapy or if I’ve entered menopause. I’m in the dark about my condition, and that’s a barrier for me; I can’t even consider pregnancy. Currently, I’m unaware of my health status, which is clearly problematic. During treatment, the side effects, including reduced libido and painful intercourse, make it challenging. The lack of a conducive atmosphere complicates matters for both the patient and their partner, requiring patience and a gentle approach*.”

Despite their experiences and the uncertainty of their clinical conditions, some survivors desire to become parents again but hesitate due to the fear that their health might deteriorate, potentially causing distress for their future children and placing a burden on their partner. João expressed: “*Some time ago, we contemplated adoption, but it’s a complex process. Even though X. is thriving with an excellent career, my health and her ability to be there for both the child and me are concerning. If a serious issue arose, her potential role as a single mother, given her career obligations, would pose significant management challenges*.” Mafalda shared: “*I dream of having two or three children and living happily ever after, but that demands stability. At present, I don’t even consider it… For me, the idea of having children is not an option. I’m relieved I don’t have any! It wouldn’t be right for a child to endure a mother constantly in the hospital, perpetually at death’s door. The constant fear of losing their mother is something I wouldn’t want for my child, nor would I want to be such a mother.*”

Fertility concerns are a significant emotional challenge for some survivors, as treatments can result in infertility, and the possibility of having children after treatment often brings sadness and regret. Despite these struggles, some survivors still desire to become parents, but fear of health deterioration and the potential burden on their families can make this goal seem unfeasible.

### The challenges of disclosure and intervention

Regarding sexuality, individuals often find it challenging to discuss their feelings with healthcare professionals. This difficulty arises partly from uncertainty about how to communicate such information and partly because these issues are deeply personal, necessitating a trusting relationship with the professional. Despite these challenges, survivors recognize the importance of seeking tangible solace through the professional’s involvement in this private aspect of their lives to discover the most effective strategies for overcoming these issues. We can interpret this from the following statements of Isabel, Matilde, and Raquel:

Isabel: “*I had always assumed the endocrinologist would be in charge of monitoring me, especially since she said she would. I attempted treatment but quickly abandoned it, and surprisingly, my partner was content. Menopause often diminishes that desire, you know*.”

Matilde: “*There’s another aspect that warrants distinct attention – our sexual and intimate lives are not discussed enough, yet I believe they’re significant*.”

Raquel: “*Since I didn’t have any professional support, what did I do?! I bought technical books, more technical sex books, no Grey’s Shadows or anything like that, because that just turns you off. Technical sex books that also give you some tips on sensuality, but above all they teach you how your body works, how a man’s body works, because in a sexual relationship there are two bodies that have to be managed, and it helped me a lot. What’s more, they also have the good sense to include tips, seduction tips for ourselves, also to pamper ourselves and not only that, they influence us to get to know our bodies in order to know what we like. That’s where I found help*.”

Many survivors face challenges discussing their sexuality with healthcare professionals, partly due to uncertainty about how to communicate these deeply personal issues and the need for a trusting relationship with their providers. Despite this, survivors acknowledge the importance of professional involvement in addressing sexual concerns, as they seek effective strategies to manage these issues, with some, turning to alternative resources, such as books and self-guided learning, to better understand their bodies and improve their intimate lives.

## Discussion

The findings of this study reveal the complex and multifaceted nature of the experiences of individuals who have undergone hematopoietic stem cell transplantation (HSCT). The participants shared that their post-transplant lives were marked by significant disruptions to both their sexuality and fertility, which were deeply intertwined with their physical, emotional, and relational well-being. Central to these disruptions is the concept of *comfort*, which emerges in different forms—physical, emotional, and psychological—and plays a critical role in survivors’ ability to navigate their post-transplant lives. This discussion explores how the presence or absence of comfort shapes survivors’ experiences and how healthcare providers can use this concept to improve the care and support they offer.

A central theme in the study was the profound disruption in sexual functioning that many survivors experienced. This disruption included reduced libido, erectile dysfunction, vaginal dryness, and painful intercourse. These physical challenges created significant barriers to intimacy and comfort, both within survivors’ relationships with themselves and with their partners. Comfort in sexual activity is often taken for granted in the general population, but for survivors of HSCT, it becomes something they can no longer rely on, as painful or difficult sexual intercourse, compounded by other physical issues like osteonecrosis or GVHD, directly interferes with the ability to experience pleasure and intimacy in relationships.

Emotional comfort also plays a pivotal role in this experience. Survivors frequently expressed feeling disconnected from their bodies as they navigated changes that disrupted their sense of sexual identity and self-worth. The emotional distress that accompanied the loss of comfort in sexual activity often led to feelings of guilt, frustration, and even shame, as expressed by participants like Alice and João. For these survivors, discomfort extended beyond the bedroom, negatively impacting their interactions with their partners and making it difficult to restore emotional closeness. As survivors sought comfort in both their bodies and their relationships, it became clear that providing emotional reassurance and support was vital in helping them regain intimacy.

Comfort within relationships emerged as a key aspect of this dynamic. Many survivors noted how their relationships with partners shifted, especially when roles transformed due to illness. The transition from being romantic partners to becoming caregiver and care-receiver often resulted in the loss of sexual and emotional comfort. This shift was evident in the experiences of Raquel and Santiago, who found that the caregiver role introduced emotional distance, which made intimacy more challenging. For these couples, achieving comfort required renegotiating their roles and finding new ways to emotionally and physically connect, even if sexual intimacy was no longer immediately attainable. These findings align with Shartau et al. [18], where participants similarly described struggles in redefining their sexual relationships after transplant, often encountering feelings of frustration or guilt. The search for a new normal post-transplant, according to Shartau et al. [18], was not always directly related to sexual health issues, but rather involved adjusting relational expectations, redefining personal boundaries, and navigating other psychosocial challenges.

Interestingly, while some survivors in our study noted the fracturing of relationships post-transplant, this issue was not easily explained. In contrast, Langer, Yi, Storer, and Syrjala [19] found that the majority of marriages survived the transplant and recovery process, with most spouses demonstrating resilience and adjusting to changes in their marital dynamics. However, female spouses were found to be more vulnerable to decreases in relationship satisfaction. As survivors like Raquel and Santiago reflect, the quest to rebuild intimacy post-transplant often involves adjusting to the emotional aftermath of HSCT and finding comfort in an evolving relationship dynamic.

Fertility concerns were another critical aspect that impacted the comfort of survivors, with fertility preservation being a particularly significant issue. Many survivors expressed regret over their inability to preserve fertility before undergoing treatment, with timing and immediate treatment needs often preventing them from pursuing fertility preservation options. Fertility loss, therefore, represents not only a physical disruption but also a significant emotional loss, as survivors like Camila and João described when reflecting on missed opportunities for parenthood.

In these cases, comfort is intertwined with a sense of regret and grief. Camila’s decision to prioritize her career over fertility preservation illustrates how external factors often influence fertility decisions, and the longing for the comfort of having been able to preserve fertility persisted in her narrative. Similarly, João’s lament over missed opportunities for fertility preservation highlights how feelings of survivor guilt and infertility grief can deeply impact emotional well-being. This aligns with findings by Liang et al. [20] and Kotronoulas, Papadopoulou, and Patiraki [21], who explored how infertility, as a treatment side effect, triggers distress and depression among survivors. The psychological burden of infertility can exacerbate sexual dysfunction and relational strain, as evidenced in our study.

The regret and anxiety surrounding fertility preservation choices are not isolated to our study participants. As shown in Mosher, Redd, Rini, Burkhalter, and DuHamel [22], a quarter of HSCT survivors experienced moderate to high levels of concern about infertility, with the majority of those under 40 expressing particularly high levels of distress. This was further supported by Hayden et al. [23] and Shanklin, Snowden, and Greenfield [24], who discussed the depressive feelings often associated with infertility. Our participants also reflected on the uncertainty surrounding their reproductive health, which further compounded their discomfort. The lack of clear answers and support in this area led to heightened anxiety about the ability to conceive in the future, mirroring the concerns of survivors like Anita and Matilde in our study. This uncertainty highlights the need for healthcare providers to offer consistent and comprehensive information about reproductive health, fertility preservation, and the potential for family planning.

Moreover, the time-sensitive nature of fertility preservation underscores the importance of timely interventions. Many HSCT patients are faced with a narrow window to decide on fertility preservation, and the urgency of starting chemotherapy often conflicts with the time needed for procedures like egg freezing [25, 26]. The delay in offering fertility preservation options can leave survivors with feelings of regret, as they realize they were not given adequate opportunities to preserve their fertility. Survivors expressed frustration over the lack of attention given to fertility concerns by their multidisciplinary care teams, emphasizing that fertility-related support is crucial in supporting survivors’ overall well-being and identity.

Beyond the physical and fertility-related challenges, survivors in our study emphasized the need for holistic comfort—encompassing emotional, psychological, and relational well-being. As survivors adjusted to their new lives after HSCT, they described a deep need to rebuild their self-esteem, body image, and relational dynamics. Raquel’s turn to educational resources like sex books to regain comfort in her body and sexuality illustrates the importance of providing accurate information that empowers survivors to take control of their bodies and sexual identities. Comfort, in this case, is not only about alleviating physical discomfort but also about rebuilding confidence and self-understanding in the aftermath of illness and treatment.

Equally important, many survivors spoke about the need for compassionate healthcare professionals who could offer reassurance, empathy, and guidance in addressing the emotional fallout of the transplant process. A consistent finding across survivors’ narratives was their frustration with the lack of professional support in addressing sexuality and fertility concerns. As Isabel and Matilde noted, the absence of open conversations around these issues left them feeling disconnected from their healthcare providers, preventing them from accessing the comfort they needed. This gap in care underscores the necessity of integrating more comprehensive psychosocial and sexual health support into post-transplant care.

Creating a supportive environment for survivors to discuss sexual and reproductive health concerns openly is essential. Healthcare providers must increase their awareness of the importance of addressing these topics and incorporate them into the holistic care process. By prioritizing comfort in discussions of sexuality, healthcare providers can foster an environment where survivors feel more comfortable engaging in open dialogue, leading to better care and improved quality of life.

In conclusion, the concept of comfort emerges as a vital element in the post-transplant experiences of survivors. Both the physical disruptions to sexuality and fertility, as well as the emotional and psychological consequences of these changes, underline the central role of comfort in the recovery process. Survivors’ struggles with intimacy, body image, and reproductive health demand holistic care that addresses not just the physiological aspects of recovery but also the emotional and relational dimensions. By focusing on comfort—through professional support, education, and empathetic care—healthcare providers can help survivors regain a sense of control over their lives and improve their post-transplant quality of life. Addressing issues of sexuality and fertility as part of holistic care is essential to supporting survivors in navigating the profound disruptions they face after HSCT.

## Strengths and limitations

This study allows unveiling a necessary phenomenon such as comfort in a population which has recently increased in number and requires nursing intervention. A limitation of this study is the fact that it was conducted in only one hematopoietic cell transplantation unit and that the results cannot be generalized. The limitations of the study can be addressed in several key areas. As this study likely involved a limited number of participants, the findings may not be broadly generalizable to all survivors of hematopoietic stem cell transplants. The unique experiences of the participants in the study may not fully represent the diverse experiences of all survivors. The reliance on self-reported data is another limitation. Participants’ accounts of their experiences are subjective and influenced by their perceptions, memories, and emotional states at the time of the interview. While the phenomenological approach values these personal experiences, the data may be impacted by participants’ ability or willingness to fully disclose sensitive information about sexuality and fertility. The findings may also be influenced by the cultural, social, and geographical context in which the study was conducted. Participants from different cultural backgrounds may have varying attitudes toward sexuality, fertility, and illness, which can impact their experiences and interpretations. These contextual differences may limit the transferability of the findings to different cultural settings or populations. While the study provided rich descriptions of the participants’ lived experiences at a particular moment in time, there may be limitations regarding long-term follow-up. Over time, individuals’ experiences of sexuality, fertility, and intimacy may change, especially as they progress through recovery or encounter new challenges. A longitudinal perspective might have provided a more complete understanding of how these aspects evolve over the course of survivorship. Even though the study aimed for rigor, the process of interpreting participants’ narratives could be influenced by researcher bias. Phenomenological analysis involves interpreting subjective experiences, and researchers’ own perspectives and experiences might unintentionally shape how they interpret participants’ accounts. The participants’ characteristics (such as age, gender, and type of treatment) could impact the diversity of experiences captured in the study. If the study includes a relatively homogenous group of participants, it may not fully capture the breadth of experiences that different individuals might have following hematopoietic stem cell transplantation.

## Conclusion

Understanding the lived experience of intimacy, sexuality, and fertility comfort of the survivor to ASCT allowed immersing in what is the meaning and what acquires meaning for people who live this time.

We can see from the descriptions of the phenomena experienced that each physical symptom is interpreted in the relationship with the partner, becoming a discomfort reflected in the relationship with the other. There is a new body after ASCT and a new way of experiencing intimacy, sexuality, and fertility. The new meaning of life leads to a redefinition of roles in couples and expectations for the future. This is an area in need of intervention and accompaniment so that intimacy goes beyond the walls of each relationship and becomes the domain of intervention by health professionals.

It prompts health professionals to consider the importance of understanding these personal experiences to reevaluate holistic care practices and address the unique needs of each individual undergoing ASCT.

## Clinical implications

With the results of this study, we were able to understand how physical changes such as pain, cGVHD, impotence, and altered libido are interpreted in relation to comfort with oneself and one’s partner. We realize that these are aspects that interfere with the sense of accomplishment, self-image, and self-perception. These are aspects that cause discomfort with oneself and guilt about not being able to fulfill one’s desires and those of one’s partner.

At the same time, we realize that these are aspects that remain a non-priority in care, something that is silenced by survivors and hidden from the focus of care by health professionals. Rethinking intervention in this area is important.

We understand that fertility preservation is still something that is put aside when treatment for an oncological disease begins. We must rethink priorities and give importance to fertility preservation and streamline these processes before starting therapeutic protocols.

Based on the findings from the study, several practical strategies can be identified to address issues of fertility and sexuality with survivors of hematopoietic stem cell transplantation (HSCT). These strategies can enhance the quality of care by providing a more holistic, patient-centered approach that acknowledges the emotional, physical, and psychological dimensions of survivors’ lived experiences.

1. Fertility preservation and family planning counseling
  Proactive fertility counseling: One of the major concerns identified by participants is the impact of treatment on fertility. Survivors often regret not addressing fertility preservation prior to treatment. Healthcare providers should implement proactive fertility counseling for all individuals prior to HSCT. This should include discussions about the potential risks to fertility, available preservation options, and the possibility of future parenthood.
  Post-treatment fertility monitoring: After treatment, regular follow-up consultations with a fertility specialist or reproductive endocrinologist could help monitor and address any changes in reproductive health. Offering fertility testing after the transplant can provide clarity for those considering parenthood in the future.
2. Addressing sexuality and intimacy concerns
  Tailored sexual health counseling: Many participants expressed challenges in their sexual lives due to physical pain, changes in libido, and body image concerns. Survivors may benefit from tailored sexual health counseling that acknowledges the diverse emotional and physical aspects of sexuality post-transplant. This counseling should provide coping strategies, including how to communicate openly with partners about sexuality, intimacy, and physical limitations.
  Psychosocial support for couples: As many participants described their relationships as being strained or altered due to the physical and emotional impacts of treatment, providing couples therapy or counseling can be beneficial. This could help couples navigate intimacy and restore connection after the transplant, supporting both emotional well-being and physical intimacy.
  Sexual rehabilitation programs: Offering sexual rehabilitation programs, including pelvic floor therapy, can address the physical changes survivors experience. These programs can help with issues such as vaginal stenosis, pain during intercourse, or erectile dysfunction, providing both education and hands-on therapy.
3. Psychosocial and holistic care strategies
  Mental health and emotional support: Given the emotional toll of dealing with infertility and changes in sexuality, survivors may benefit from psychological support to address grief, loss, and feelings of guilt. Mental health professionals, including psychologists and counselors, should be integrated into the care team to provide counseling related to body image, identity, and self-esteem.
  Peer support groups: Survivor-led support groups can offer a sense of community and solidarity, allowing individuals to share experiences and advice about coping with sexuality and fertility post-transplant. These groups can help reduce feelings of isolation and provide valuable insights from peers who have faced similar challenges.
  Holistic care approach: Integrating a holistic care model that addresses physical, emotional, and psychological well-being is crucial. This might include complementary therapies such as mindfulness, meditation, and yoga to help manage stress, improve body image, and foster emotional healing. By addressing the whole person rather than just the medical aspects of care, healthcare providers can enhance quality of life and promote resilience in survivors.
4. Education and information resources
  Providing information about sexual health: Many participants, such as Raquel, reported a lack of professional guidance on sexuality and intimacy. Healthcare providers should offer information and resources about sexual health post-transplant, including the potential side effects of treatment on libido, hormonal function, and physical intimacy. This could be delivered through educational materials, workshops, or one-on-one counseling sessions.
  Facilitating open communication: Encouraging survivors to have open conversations with their healthcare providers about sensitive issues like sexuality can help reduce the discomfort and stigma often associated with these topics. Ensuring that the healthcare environment is accepting and non-judgmental can help survivors feel more comfortable discussing these aspects of their lives.
5. Improving partner involvement
  Partner education: For many survivors, the challenges to intimacy and sexuality are not only personal but shared with their partners. Including partners in counseling sessions and providing them with education about the survivor’s condition, its effects on intimacy, and how to support each other can be an essential strategy. Partners may also experience emotional distress due to changes in their relationship, so offering resources to them is equally important.
  Addressing caregiver-partner dynamics: The shift in roles, where a partner becomes a caregiver, was noted by participants as complicating intimacy. Addressing this change through couples counseling or role redefinition discussions can help partners reconnect emotionally and sexually after the transplant.

By integrating these strategies, healthcare providers can offer more comprehensive, personalized care that takes into account not only the medical needs of survivors but also their emotional, relational, and sexual well-being. This approach will support the long-term recovery and quality of life for survivors, addressing the complex intersection of fertility, sexuality, and identity in the context of post-transplant survivorship.

## Data Availability

The data that support the findings of this study are not openly available due to reasons of sensitivity and are available from the corresponding author upon reasonable request.

## Abbreviations

ASCT: Allogeneic stem cell transplant
GVHD: Graft-versus-host disease
